# Improving of Gelling Capacity of Cooked Crab Meat Proteins

**DOI:** 10.3389/fnut.2021.675362

**Published:** 2021-09-30

**Authors:** Gabriela Nallely Trejo-Díaz, Miguel Ángel Martínez-Maldonado, Rocío M. Uresti-Marín, Gonzalo Velazquez, José Alberto Ramírez

**Affiliations:** ^1^Unidad Académica de Trabajo Social y Ciencias para las Humanidades, Universidad Autónoma de Tamaulipas, Ciudad Vic, Mexico; ^2^Facultad de Nutrición, Universidad de Ciencias y Artes de Chiapas, Tuxtla Gutiérrez, Mexico; ^3^Centro de Física Aplicada y Tecnología Avanzada, Universidad Nacional Autónoma de México, Querétaro, Mexico; ^4^Instituto Politécnico Nacional-Centro de Investigación en Ciencia Aplicada y Tecnología Avanzada, Unidad Querétaro, Querétaro, Mexico

**Keywords:** crab meat, microbial transglutaminase, mixing, cutting, gelling

## Abstract

Cooked crab meat subjected to a cutting process can aggregate again, forming weak gels. The objective of this work was to determine the effect of two mixing methods, combined with the addition of the microbial enzyme TGase (MTGase) on the mechanical and functional properties of gels from washed or unwashed blue crab (*Callinectes sapidus*) meat. Live crabs were obtained from Laguna Madre, Tamaulipas, Mexico, and cooked at 120°C for 20 min before hand-picking the meat from the shell. Cooked meat was processed by mixing and cut at temperatures of 25 or 60°C, without (control) or 0.5% of MTGase. Then cooked at 90°C for 15 min. Changes in texture profile analysis, percentage of extractable water, and color were evaluated. The mixing method at 60°C allowed increasing the textural properties of the gels, and the addition of MTGase significantly improved the mechanical properties. The results allowed stablishing a viable technique to obtain restructured gels from cooked crab meat with no need to extract the soluble compounds responsible for their distinctive odor and taste which often affect the mechanical properties.

## Introduction

The crabs belong to the brachyury group (brachyura), as they are decapod crustaceans, and include about 4,500 species (1). They are the livelihood of high demand and commercial value fisheries worldwide (2).

Crabs are immersed in water at boiling or retorting temperature, to facilitate the manual extraction and separation of meat. Each anatomical section has different economic value in the international market. This cooking process limits the production of restructured crab products, as cooked meat from vertebrates, and invertebrates do not usually form gels (3).

Obtaining restructured products from cuts of meat from vertebrates and invertebrates requires solubilizing the native muscle proteins with salt, homogenizing, formatting, and cooking to obtain the desired shape (4). Meat must not have been previously heated or frozen to obtain quality products (5).

The frozen Johan crab cooked meat (*Cancer borealis*) can form weak gels if washed with cold water (a technique used to obtain surimi) and does not require the use of salt to gel when incubated at 35°C for 30 min before cooking it to 90°C for 30 min (6, 7). The authors did not mention the effect that washing cooked crab meat has on taste as this process removes water-soluble compounds associated with the taste, smell, and color of fish meat and should have the same effect on crab meat.

Blue crab meat (*Callinectes sapidus*) cooked at 120°C had better textural characteristics than meat obtained by cooking at 50–70°C, suggesting that the gelling mechanism of cooked meat is associated with the unfolding of previously thermally aggregated proteins (8). Adding microbial transglutaminase improved the properties of cooked crab gels requiring only one cold water washing cycle to obtain restructured products (9, 10).

The use of high hydrostatic pressures at 400 MPa for 5 min improved the mechanical properties of thermally induced fish gels; however, after raising the pressure level to 600 MPa, the increase in texture was less (11). In gels obtained from cooked crab meat, a favorable effect of the pressure was observed on the textural properties when the gels were pressurized to 100 MPa. This effect was less important when pressures of 300 or 600 MPa were used (12).

Studies on cooked crab meat have allowed developing processes to obtain gels from muscle proteins shredded to fine grain. Still, these products have a weak texture, compared to commercial restructured products. The objective of this work was to develop a process that would improve the mechanical properties of gels, considering the particle size of the matter used in the process and the effect of adding transglutaminase to washed or unwashed crab meat.

## Materials and Methods

### Crab Meat

The blue crab was captured in Laguna Madre, in the vicinity of the town Carboneras, located in the municipality of San Fernando, Tamaulipas, Mexico. The crabs were transported to the facilities of a processing plant located in the city of San Fernando, Tamaulipas, Mexico, within the first 4 h after its capture. The blue crab was processed at 120°C for 20 min in a commercial autoclave and the meat was removed from the shell manually on the production line. The crab meat was stored in plastic containers with abundant ice and transported to the lab for further processing.

### Washing Crab Meat

The cooked crab meat was processed between 4 and 6 h after being received in the laboratory. The meat underwent a washing cycle with cold water below 4°C, in a ratio of 3:1 water/meat, stirring gently for 7 min, and letting the mixture rest for 10 min before draining. A commercial fabric was used to manually extract the excess water.

### Production of Crab Meat Gels

Cooked crab meat gels were obtained from unwashed or with a wash cycle meat. Two processes were used: mixing or cutting. The mixing process was carried out using a commercial 6-speed blender (Black & Decker, model MX3200B, Mexico). The ingredients were dispersed homogeneously into the meat for 3 min, at speed 3, using the beater blades included in the equipment. The microbial TGase (MTGase, Active TG-TI, Ajinomoto USA, Inc., Teaneck, NJ) was added in powder, at 0.5% w/w, and two mixing temperatures were used: 25 or 60°C. An MTGase-free control gel was obtained for each treatment. To prepare gels by the chopping process, a Hamilton Beach cutter of 0.7 L (model 72860, USA) was used, cutting the samples for 1 min at 25 or 60°C. MTGase was added at 0.5% w/w. Control gels were obtained without MTGase. Mixed or cut pastes were introduced into stainless steel tubes (1.8 cm inner diameter, 17.7 cm in length). The steel tubes were closed with threaded plugs before incubation at 60°C for 30 min, followed by a water immersion at 90°C for 15 min. After cooking, the tubes were placed in a cold-water bath (5°C) for 30 min and stored for 10 h at 4°C.

### Texture Profile Analysis

The mechanical properties were determined following the method described by Martínez et al. (8), using a Stable Micro Systems Texturometer (Model TAXT2i, Vienna Court, England, UK). The size of the gel samples was 1.87 cm in diameter and 3 cm in length and equilibrated at room temperature by 30 min, in plastic bags, to avoid dehydration before measurements. Texture profile analysis (TPA) was performed by compressing samples at 75% of their initial height. A cylindrical aluminum probe (P/50) with a 50 mm diameter and a crosshead speed of 60 mm/min were used. The hardness, cohesiveness, springiness, and chewiness values were reported. Six samples were analyzed for each treatment.

### Color

A precise color reader portable colorimeter (HP 2000, Guangdong, China) calibrated with a white and black tile, identified as standards, was used to assess color in gels. Six replicates were performed on each sample (six control samples and six samples with a washing cycle for mixed or cut pastes). The parameters obtained by the instrument (L^*^, a^*^, and b^*^), were used to calculate the values of C^*^ and H^*^.

### Expressible Water

The expressible water content was determined by centrifugation. Restructured samples (5 g) were wrapped in Whatman No. 1 paper, with a size of 15 × 15 cm. A triplicated determination was made for each of the treatments. The initial weight of each sample was recorded. The wrapped samples were placed in a centrifuge (Hettich Zentrifugen Rotofix 32-A, Tuttlingen, Germany) at 1000 rpm for 5 min. The final weight of the sample was recorded, and the removable water was determined.


(1) $$\begin{array}{r} Expressible\;water={\frac{\;Initial\;weight-final\;weight}{Initial\;weight}}^{*}100 \end{array}$$

### Statistical Analysis

A multifactorial analysis of variance was carried out using Statgraphics 5 software (Manugistics, Inc., Rockville, MD). Differences between mean values were established using the least significant difference (LSD) multiple range test and they were considered significant when *p* < 0.05.

## Results and Discussion

The effect of the particle size (mixing or cutting) of crab meat on the textural properties of gels was analyzed. The structure of the fibers remained unaltered in the mixing batch, meanwhile, the other batch underwent a considerable reduction by cutting. In both cases, no salt was added, as it has been reported that it is not required to solubilize proteins when obtaining crab gels (7). This property suggests that, unlike other restructured meat products, shredding may not be required either to obtain gels from crab meat. Changes induced by adding microbial transglutaminase enzyme, subjecting meat to cold water washes, and mixing type and temperature were also evaluated.

### Hardness

**Figure 1:**
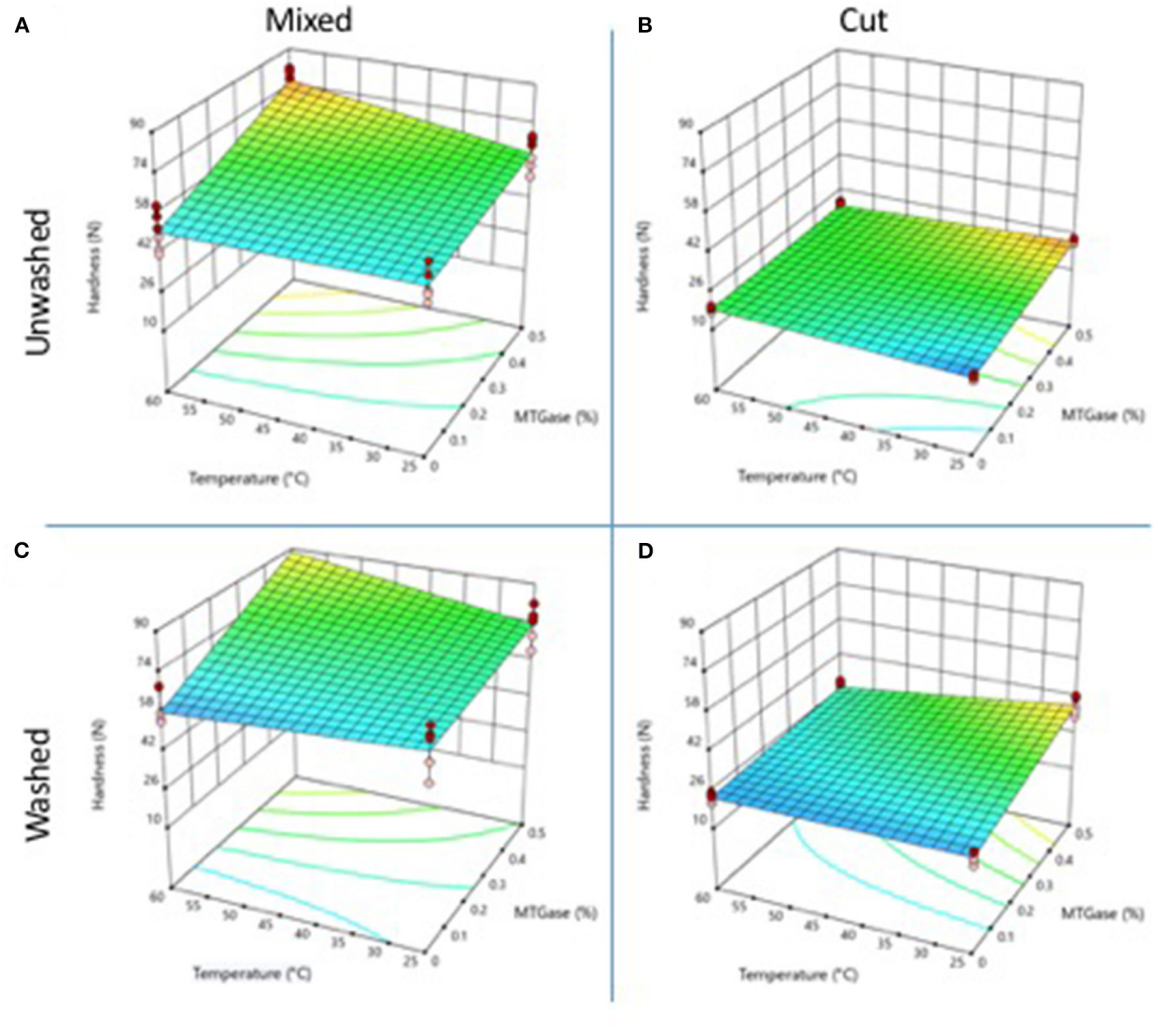
Changes in gel hardness properties as a function of the type and temperature mixing method, washing of the cooked meat, and adding of MTGase. **(A)** Mixed-unwashed samples, **(B)** Cut-unwashed samples, **(C)** Mixed-washed samples, and **(D)** Cut-washed samples.

Figure 1A shows the behavior of the hardness values of gels obtained from unwashed crab meat. In control gels, without the addition of microbial TGase, it was observed that the mixing treatment allowed obtaining gels with hardness values of 51.80 and 50.95 N at 25 and 60°C, respectively, significantly higher (*p* ≤ 0.05) than those obtained by the cut treatment (15.25 and 18.67 N at 25 and 60°C, respectively) (Figure 1B). No significant difference (*p* ≤ 0.05) was found by the effect of the mixing temperature of 25 and 60°C. The incorporation of 0.5% of microbial TGase allowed increasing hardness values in both treatments (Figures 1A,B). Microbial transglutaminase (MTGase) is a calcium-independent enzyme, which can be obtained from *Streptoverticillium ladakanum* or *Streptoverticillium mobaraense*. This enzyme catalyzes an acyl transfer reaction between γ-carboxiamide groups of glutaminyl residues in proteins. When the primary amine is the ε-amino group of lysine and lysil residues, ε-(γ-glutamyl) lysine cross-linking is formed. The enzyme is commercially used to improve the gelling capacity of different meat proteins (13). The effect was significantly higher (*p* ≤ 0.05) on gels obtained by mixing and subjected to 60°C, with hardness values of 76.25 N. In cut gels, MTGase improved hardness when cutting at 25°C, although, at 60°C the enzyme had no effect. Cooked crab meat forms weak gels if soluble proteins are not extracted by washing or pressing (6, 7). Cooked meat from Chinese mitten crab (*Eriocheir sinensis*) decreased the mechanical properties of surimi gels when added at 10–30% into surimi gels (14). Mechanical properties of gels from cooked crab meat, obtained by the cutting process, increased by incubating them at 40°C for 30 min with MTGase before cooking at 90°C (8). In this work, cooked crab meat showed high hardness values just by mixing the whole muscle fibers (without cutting) at 60°C before cooking directly at 90°C without previous incubation.

The gels obtained by washing the cooked meat with cold water, to remove the water-soluble protein partially, had higher hardness values than those obtained from unwashed meat (Figure 1). Control gels obtained without MTGase, using the mixing technique (Figure 1C), had higher hardness values than those of gels obtained by cutting. In both processes (mixed and cut) no significant difference (*p* ≤ 0.05) was found by processing temperature (Figures 1C,D). The addition of MTGase increased the hardness value in all gels. The gels obtained by mixing at 60°C had the highest hardness value (88.60 N).

Washing the cooked crab meat three times increases the mechanical properties of the gels but also eliminates the distinctive odor and flavor from crab. The addition of MTGase to washed cooked crab meat significantly improves the final texture (8). It is possible to apply a single wash cycle to the crab meat and combine it with MTGase to improve the mechanical properties, however, the obtained gels are weak and do not have the same textural properties as the commercial restructured products (3).

### Cohesiveness

**Figure 2:**
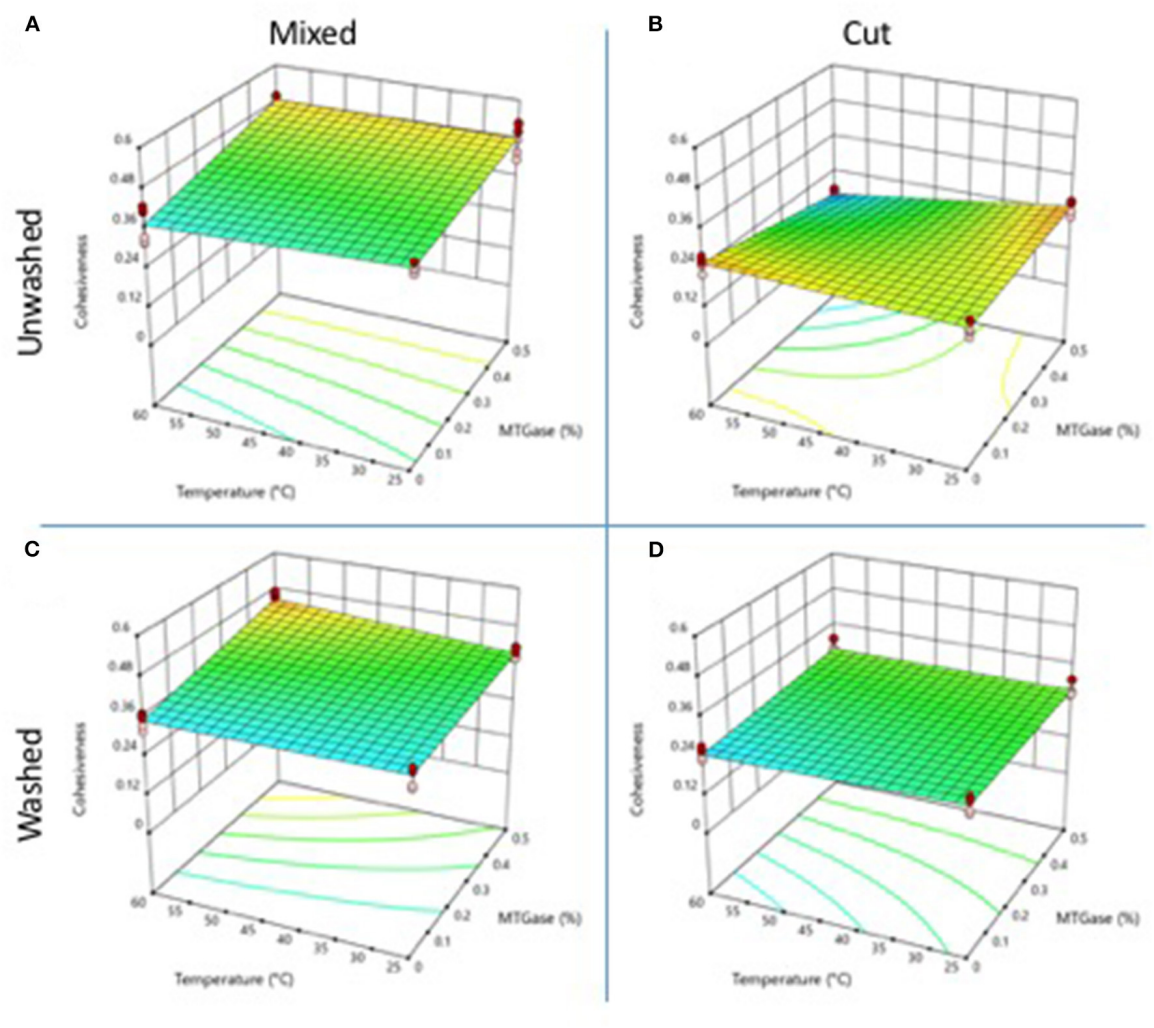
Changes in gel cohesiveness properties as a function of the type and temperature mixing method, washing of the cooked meat, and adding of microbial MTGase. **(A)** Mixed-unwashed samples, **(B)** Cut-unwashed samples, **(C)** Mixed-washed samples, and **(D)** Cut-washed samples.

Figure 2 shows the cohesiveness of gels obtained from cooked crab meat. The gels obtained by mixing (Figure 2A) had significantly higher values (*p* < 0.05) than the gels obtained by cutting (Figure 2B). The gels obtained by mixing, with no MTGase, showed a cohesiveness value of 0.40 at 25°C and 0.40 at 60°C. In gels obtained by cutting, the cohesiveness was 0.23 and 0.25 for 25 and 60 °C, respectively. The low cohesiveness values in gels obtained by cutting indicate that the sample lost its internal structure during the first compression, so it did not require much force to compress it again. In gels obtained by mixing, the presence of the fibers of the crab muscle induced a more cohesive structure. The addition of MTGase allowed increasing the cohesiveness values. The gels obtained by mixing at 25°C had a value of 0.48, while, in gels processed at 60°C, it was 0.54. In gels prepared by cutting, no effect of MTGase was observed, with cohesiveness values of 0.23 and 0.16 at 25 and 60°C, respectively. Washing with cold water did not have much effect on cohesiveness values (Figures 2C,D). Gels from unwashed cooked crab meat, obtained with 0.5% MTGase and mixed at 60°C, showed the highest cohesiveness values.

### Springiness

**Figure 3:**
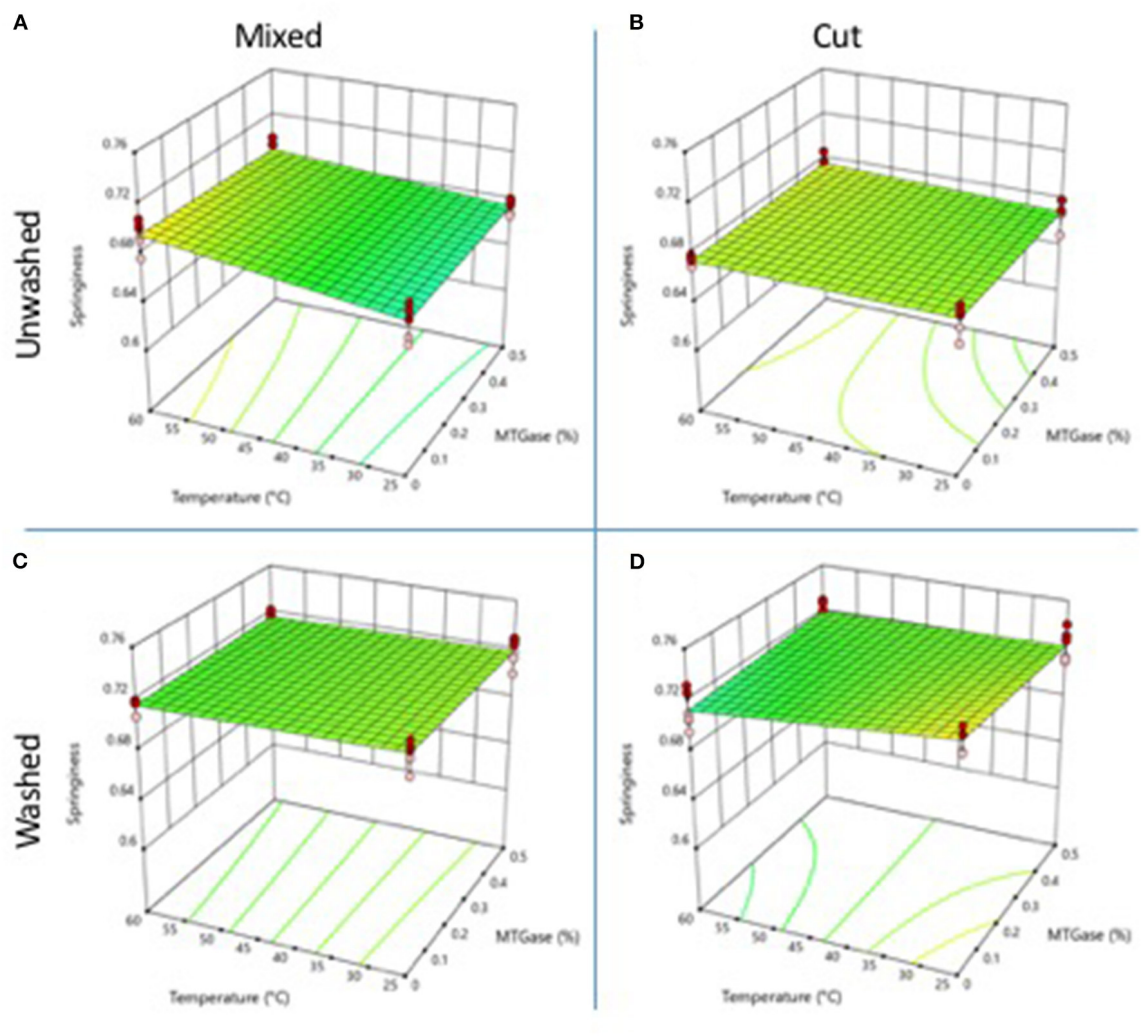
Changes in gel springiness properties as a function of the type and temperature mixing method, washing of the cooked meat, and adding of microbial MTGase. **(A)** Mixed-unwashed samples, **(B)** Cut-unwashed samples, **(C)** Mixed-washed samples, and **(D)** Cut-washed samples.

The springiness of the gels was not significantly affected (*p* ≤ 0.05), by none of the treatments (Figure 3). Springiness values ranged from 0.67 to 0.68 in gels obtained from unwashed meat (Figures 3A,B). The gels obtained from cooked and washed meat had slightly higher springiness values (Figures 3C,D), ranging from 0.71 to 0.72, although, this difference was not significant (*p* < 0.05). Springiness is a texture parameter that usually remains at intermediate values and is not modified by washing or the use of MTGase (3). In the present work, this parameter was neither increased nor diminished by the mixing technique.

### Chewiness

**Figure 4:**
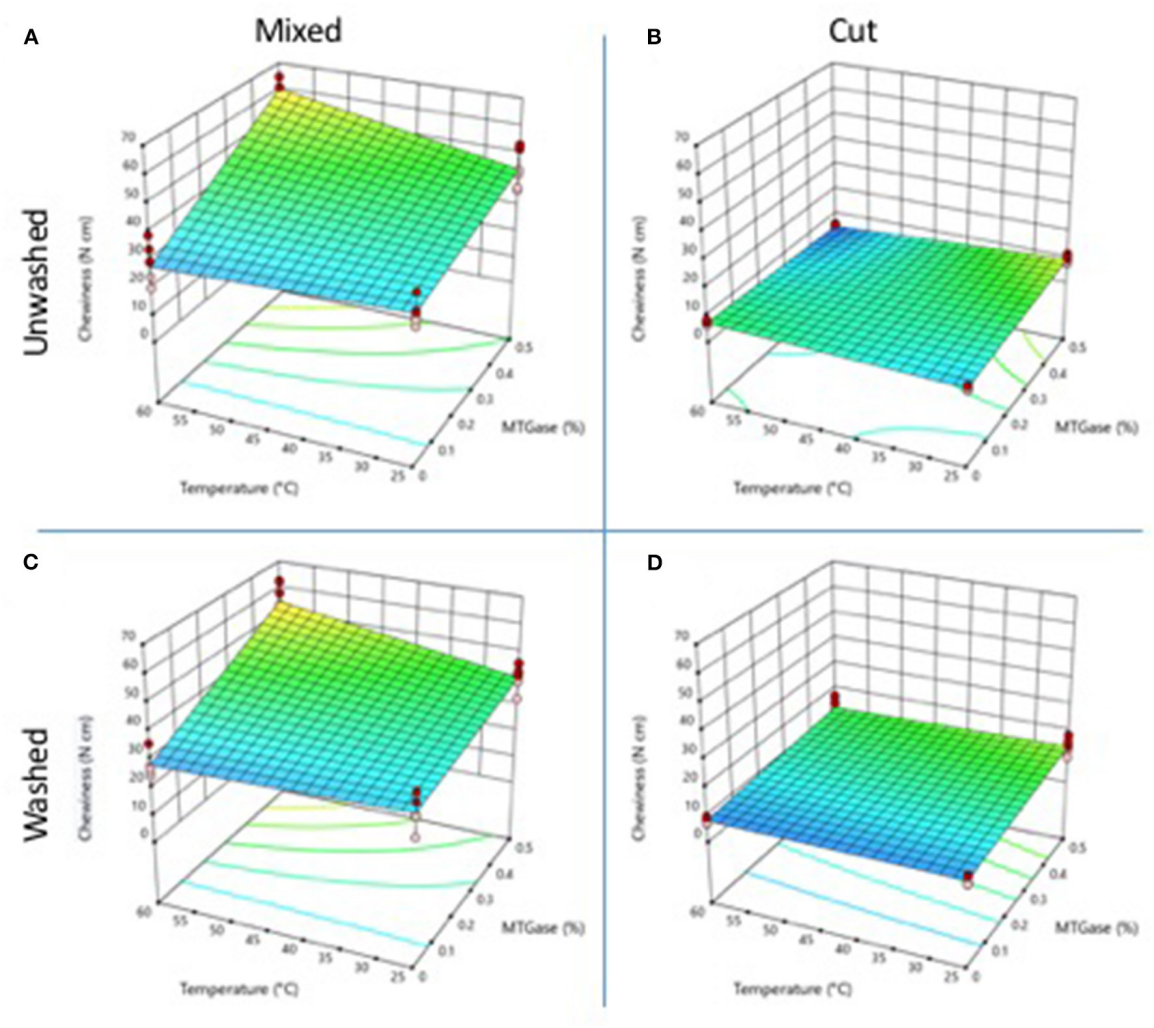
Changes in gel chewiness properties as a function of the type and temperature mixing method, washing of the cooked meat, and adding of microbial MTGase. **(A)** Mixed-unwashed samples, **(B)** Cut-unwashed samples, **(C)** Mixed-washed samples, and **(D)** Cut-washed samples.

Chewiness represents the energy required to chew a solid food and disintegrate it to swallow it. Figure 4 shows the chewiness values. Gels obtained from cut crab meat (Figures 4B,D) showed lower values of chewiness than gels from mixed crab meat.

The mixing temperature (25 or 60°C) did not significantly change (*p* ≤ 0.05) the chewiness of gels obtained from unwashed and MTGase-free cooked crab meat, presenting values of 31.35 and 27.77 N cm, respectively. The gels obtained by the cutting process, showed lower chewiness values (*p* ≤ 0.05) than those of mixing, being 5.29 and 7.14 N cm, respectively.

The addition of 0.5% MTGase increased the chewiness of the gels obtained by mixing, but not in the gels obtained by cutting. The effect was higher on gels obtained from unwashed crab meat, at 60°C, reaching a value of 60.02 Ncm (Figure 4A). This value was slightly higher than that of gels obtained by cutting (54.62 N) at the same temperature with washed cooked meat (Figure 4C). The chewiness is a textural parameter that is favored by the addition of MTGase under incubation conditions, in which the enzyme is allowed interacting and forming covalent bonds between adjacent protein chains (9). In the present work, even without incubation, MTGase improved the chewiness of gels obtained by mixing at 60°C.

### Extractable Water

**Figure 5:**
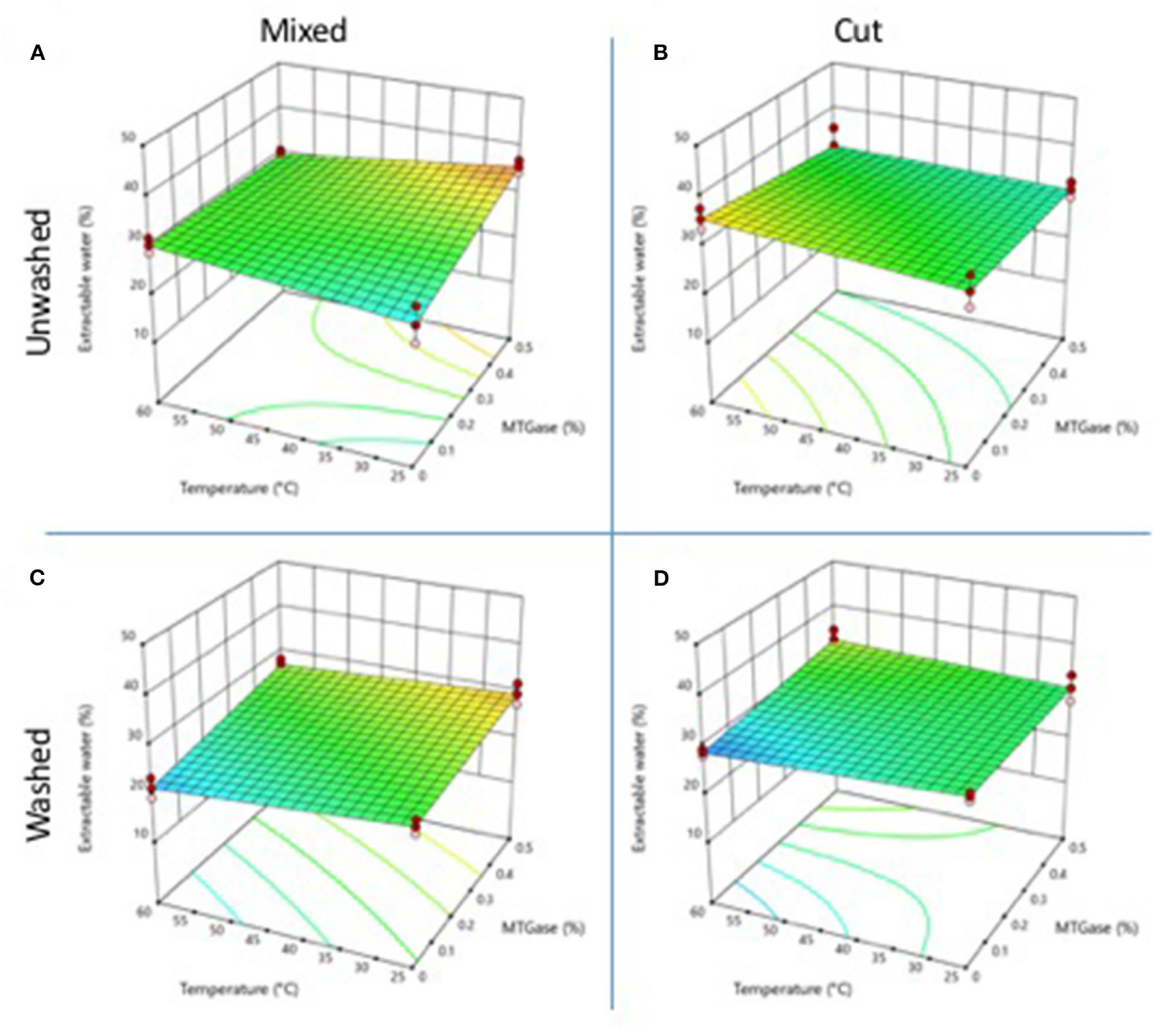
Changes in gel extractable water properties as a function of the type and temperature mixing method, washing of the cooked meat, and adding of MTGase. **(A)** Mixed-unwashed samples, **(B)** Cut-unwashed samples, **(C)** Mixed-washed samples, and **(D)** Cut-washed samples.

Extractable water content (EW) is inversely associated with water holding capacity (WHC). Low values of extractable water mean high WHC. Gels obtained from unwashed meat, by the mixing method with no MTGase, showed EW values of 25.48 and 30.11% at 25 and 60°C, respectively (Figure 5A); while gels obtained by the cutting process (Figure 5B) had higher EW values of 32.15 and 35.52% for 25 and 60°C, respectively. The addition of 0.5% of MTGase increased the value of EW in gels mixed at 25°C (37.57%) but not in the gels prepared by mixing at 60°C. In gels obtained by the cutting process, the addition of 0.5% of MTGase decreased significantly (*p* ≤ 0.05) the EW values at both temperatures.

Gels obtained from meat washed with cold water, by mixing at 60°C with no MTGase, presented lower values of EW than gels processed at 25°C (Figure 5C). The addition of 0.5% of MTGase resulted in higher values of EW. The gels obtained by cutting had higher EW values than those of gels obtained by mixing (Figure 5D). Gels obtained by mixing or cutting showed EW >25%, indicating that additional strategies are required to improve the water holding capacity, such as the use of gums, starches, or other hydrocolloids. High levels of EW have been previously reported in gels obtained from cooked crab meat (7, 8).

### Color Attributes

Table 1A shows the changes in color of unwashed cooked crab meat gels. In gels obtained without MTGase and by mixing process, the luminosity values (L) were 74.6 and 74.1, Chroma (C) of 4.8 and 4.5, and Hue (H) of 130.7 and 125.8 at 25 and 60°C, respectively. These values are located at the grayish-green hue region of the color scale. Gels obtained by the cutting process showed L^*^ values of 80.03 and 77.78, C^*^ values of 4.3 and 4.5, H^*^ of 74.49 and 83.3 at 25 and 60°C, respectively. These values correspond to grayish-yellow hues. Crab meat gels from cooked meat with 0.5% MTGase prepared by mixing had values similar to those obtained in enzyme-free treatments: 75.9 and 74.0 for L^*^, 5.2, and 4.5 for C^*^ and 123.6 and 125.5 for H^*^, at 25 and 60°C, respectively. These values correspond to grayish-yellow hue region. The gels obtained from washed meat had color attributes very similar to gels obtained from unwashed meat (Table 1B). The small changes in color attributes might be because the crab meat is pale, not intense, and was only washed with cold water, which prevented the extraction of several compounds responsible for the color.

**Table 1:** Changes in color attributes of gels by the effect of the type and temperature mixing method, washing of the cooked meat and adding of microbial TGase.

**Color atribute**	**25** ^**°**^ **C**				**60** ^**°**^ **C**			
	**Control**		**0.5% MTGase**		**Control**		**0.5% MTGase**	
	**Mixed**	**Cut**	**Mixed**	**Cut**	**Mixed**	**Cut**	**Mixed**	**Cut**
**(A) Gels from unwashed cooked crab meat**								
L*	74.6 ± 1.4^a^	76 ± 0.8^a^	75 ± 1.1^a^	75.9 ± 1.1^a^	74.1 ± 0.9^a^	75.6 ± 0.6^a^	74 ± 1^a^	75 ± 1.1^a^
a*	−3.2 ± 0.8^a^	−1.9 ± 0.1^b^	−2.9 ± 0.7^a^	−1.9 ± 0.3^a^	−2.6 ± 0.3^a^	−2 ± 0.8^a^	−2.6 ± 0.7^a^	−3.4 ± 0.3^a^
b*	3.6 ± 0.4^a^	4.3 ± 0.3^a^	4.3 ± 0.8^a^	4.4 ± 0.2^a^	3.7 ± 0.6^a^	4.1 ± 0.7^a^	3.7 ± 0.6^a^	4.7 ± 0.4^a^
C*	4.8 ± 0.4^a^	4.7 ± 0.2^b^	5.2 ± 0.9^a^	4.8 ± 0.3^a^	4.5 ± 0.5^a^	4.6 ± 0.9^a^	4.5 ± 0.7^a^	5.9 ± 1.4^a^
H*	130.7 ± 8.8^a^	113.6 ± 2.8^b^	123.6 ± 4.8^a^	113.9 ± 3.5^b^	125.8 ± 6.2^a^	114.6 ± 10^a^	125.8 ± 0.7^a^	124.2 ± 11.1^a^
Whitness	74.2 ± 1.4^a^	75.5 ± 0.8^a^	75.3 ± 1.3^a^	76 ± 0.8^a^	73.7 ± 1.2^a^	75.1 ± 0.5^a^	73.6 ± 0.9^a^	74.3 ± 1.2^a^
**(B) Gels from washed cooked crab meat**								
L*	77.2 ± 1.9^a^	80 ± 0.5^b^	77.6 ± 2.4^a^	80.2 ± 1.3^a^	75 ± 0.9^a^	77.7 ± 0.4^b^	77 ± 0.7^a^	78.2 ± 0.7^a^
a*	0.3 ± 0.4^a^	1 ± 0.4^a^	−0.1 ± 1.4^a^	1.19 ± 0.6^a^	−0.8 ± 0.4^a^	0.5 ± 0.2^b^	−0.5 ± 0.3^a^	0.9 ± 0.9^b^
b*	3 ± 0.9^a^	4.1± 0.3^a^	3.1 ± 0.7^a^	4.4 ± 0.6^a^	3.1 ± 0.9^a^	4.5 ± 0.7^a^	3.8 ± 0.9^a^	4.8 ± 0.9^a^
C*	3 ± 1.0^a^	4.3 ± 0.3^a^	3.4 ± 0.5^a^	4.6 ± 0.5^b^	3.2 ± 1^a^	4.5 ± 0.7^a^	3.9 ± 0.9^a^	5 ± 0.9^a^
H*	81.4 ± 5.7^a^	75.4 ± 6.5^a^	96.8 ± 27.1^a^	74.6 ± 8.7^a^	104.9 ± 5.8^a^	83.3 ± 2.7^b^	98.2 ± 6.3^a^	78.9 ± 6.5^b^
Whitness	77 ± 1.8^a^	79.5 ± 0.5^b^	77.4 ± 2.3^a^	79.7 ± 1.1^a^	74.8 ± 3.4^a^	77.3 ± 0.4^a^	76.6 ± 0.7^a^	77.7 ± 0.6^a^

*a,bDifferent letters indicate differences (P < 0.05) between treatment (mixed or cut) for each measured property. Values indicate the means and its respective standard deviations*.

### Gelling Improving

Crabs require to be steamed or boiled to separate the meat from the shell. The cooking operation affects the functional properties of crab meat limiting its use to produce restructured products. A few studies have recently proposed extracting the soluble proteins by centrifugation (6, 7) or by a washing process, like those used in surimi technology, to improve the gelling capacity (8). Still, such processes negatively affect the unique flavor of crab, which is highly demanded by consumers. Using of MTGase has been proposed to improve the mechanical properties of protein gels from cooked crab meat, allowing reducing from three to only one the number of washing cycles needed to extract the soluble proteins, but the flavor of crab meat is affected (3). For the first time, this work proposes a novel but straightforward technique to improve the mechanical properties of gels obtained from cooked crab meat proteins. The mentioned process involves only mixing the cooked meat fibers of crab at a mild temperature to induce denaturation of proteins and obtaining a better protein network strengthened by the action of MTGase. This inner network allows obtaining gels with appropriated gelling properties and preserving the distinctive flavor of the crab. The process is simple, of low cost, technically feasible, and easy to implement. Although, MTGase decreases the water holding capacity, adding gums or starches which are extensively used in restructured products, may improve this functional property with a low adverse effect on texture or flavor.

## Conclusions

Mixing the cooked crab meat, in the absence of salt, for a short time, allowed obtaining meat paste that, when formatted and heated at 90°C induced the formation of gels with textural properties suitable for a restructured product. The addition of MTGase at 0.5% allowed increasing the mechanical properties, but decreased water holding capacity. The proposed method of mixing cooked crab meat fibers, unlike the traditionally reported cutting method, offers an alternative for producing restructured products from crustaceans, which require a previous cooking resulting in poor textural properties when using traditional techniques. The technique reported in this work offers a feasible alternative to process and commercialize crab meat.

## Data Availability Statement

The original contributions presented in the study are included in the article/supplementary materials, further inquiries can be directed to the corresponding author/s.

## Author Contributions

All authors listed have made a substantial, direct and intellectual contribution to the work, and approved it for publication.

## Conflict of Interest

The authors declare that the research was conducted in the absence of any commercial or financial relationships that could be construed as a potential conflict of interest.

## Publisher's Note

All claims expressed in this article are solely those of the authors and do not necessarily represent those of their affiliated organizations, or those of the publisher, the editors and the reviewers. Any product that may be evaluated in this article, or claim that may be made by its manufacturer, is not guaranteed or endorsed by the publisher.
